# Construction of a P/N/Zn Synergist for Enhancing the Fire Safety and Char Formation of PA6/Aluminum Diethylphosphinate Composites

**DOI:** 10.3390/polym18030351

**Published:** 2026-01-28

**Authors:** Qinghua Peng, Yifang Hua, Jingjing Yang, Yujia Wang, Gehao Guo, Wanen Li, Jun Sun, Xiaoyu Gu, Jianhua Li, Sheng Zhang

**Affiliations:** 1 State Key Laboratory of Organic-Inorganic Composites, Beijing University of Chemical Technology, Beijing 100029, China; 2 Beijing Key Laboratory of Advanced Functional Polymer Composites, Beijing University of Chemical Technology, Beijing 100029, China; 3 School of Police Equipment and Technology, Chinese People’s Police University, Langfang 065000, China

**Keywords:** PA6, flame retardant, char formation, synergist

## Abstract

Polyamide 6 is an important engineering thermoplastic; however, its practical use is often constrained by its high flammability. Although aluminum diethylphosphinate is widely employed as a flame retardant for polyamide 6, its relatively slow char-forming kinetics hinders the attainment of the stringent 750 °C glow-wire ignition temperature required for electrical applications at moderate loadings. To address this limitation, a synergist was fabricated via the self-assembly of phytic acid, benzoguanamine, and ZnSO_4_·7H_2_O and subsequently incorporated to enhance the char-forming capability and flame retardancy of polyamide 6/aluminum diethylphosphinate composites. The results revealed that the synergist acted as an efficient charring promoter, improving flame retardancy. At a total loading of 15 wt%, the composite reached a UL-94 V-0 rating and high limiting oxygen index of 30.7%. Cone calorimetry data indicate that the peak heat release rate decreased by 34.0%, and the smoke production rate decreased by 33.3% compared with the polyamide 6/aluminum diethylphosphinate composites. Mechanistic analysis indicated that the synergist catalyzed the carbonization of the polyamide 6, enabling the formation of a dense thermally insulating char barrier in the condensed phase. Notably, the optimized formulation achieved a glow-wire ignition temperature of 750 °C, demonstrating its strong potential for high-safety electrical applications.

## 1. Introduction

Polyamide 6 (PA6), one of the five major engineering plastics, has been widely used in the automotive, electrical, and aerospace fields owing to its excellent mechanical strength, thermal stability, insulation properties, and processability [1,2,3]. However, PA6 undergoes severe melt dripping and rapid combustion upon heating, and its intrinsic flammability significantly limits its application in fire-critical scenarios [4,5,6]. Therefore, the development of highly efficient flame-retardant systems suitable for PA6 is urgently required [7,8,9].

In recent years, organophosphorus compounds have been regarded as highly efficient halogen-free flame retardants. Among them, aluminum diethylphosphinate (ADP) has been widely applied in PA6 due to its excellent thermal stability and low required dosage [10,11,12]. However, the flame-retardant action of ADP is predominantly governed by a gas-phase mechanism: phosphorus-containing radicals (PO•, HPO•, HPO_2_•) generated during combustion effectively scavenge HO• and H• radicals, thereby exerting a gas-phase quenching effect [13,14,15]. As a result, its ability to promote condensed-phase char formation is limited; the charring kinetics are slow, and the resulting char is cannot serve as an efficient barrier to heat and mass transfer [16,17,18], making it difficult to satisfy the stringent electrical safety requirements such as a 750 °C glow-wire ignition temperature (GWIT). In addition, the insufficient condensed-phase contribution often leads PA6/ADP systems to display relatively high heat release and smoke production rates [19,20,21]. Therefore, the construction of more effective synergistic flame-retardant systems is needed to enhance the condensed-phase charring and reduce smoke hazards.

To address these challenges, a synergistic flame-retardant strategy has emerged as an effective approach to enhance the fire safety of PA6. In this strategy, the design of multi-component systems achieve performance beyond that of a single flame retardant by synergistic interactions [22,23,24]. Chai et al. [25] synthesized a lamellar cerium phenylphosphonate, which exhibited effective synergy with ADP in mitigating the candlewick effect of polyamide 6/carbon fiber composites. Wang et al. [26] synthesized a novel macromolecular flame retardant that, in combination with ADP, showed good synergistic flame-retardant performance in PA6 during vertical burning tests.

In such systems, phosphorus sources are commonly employed as core units. Phytic acid (PA) with a high phosphorus content (~28 wt% P) and multiple phosphate groups can promote char formation during combustion, making it a promising condensed-phase agent. Moreover, its oxidized phosphorus structures are more likely than ADP to retain stable phosphates or polyphosphates after thermal decomposition, thereby improving the char quality and barrier efficiency [27,28,29,30]. However, the inherent acidity and relatively low decomposition temperature of PA can compromise the thermal stability of the matrix if used alone, requiring its combination with other components to construct a stable flame-retardant system [31]. Nitrogen sources, such as benzoguanamine (BG), can partially neutralize the acidity through condensation reactions between –NH_2_ groups and the P–OH of PA, enhancing the thermal stability. During combustion, these nitrogen sources further release nonflammable gases, which dilute combustible volatiles and contribute to the expansion of the char layer. For example, PA6 composites containing 7 wt% PhA–MEL–MWCNTs exhibited an LOI increase from 21.0 to 26.4 and attained UL-94 V-0 classification [32]. Transition metal ions demonstrate remarkable catalytic effects during polymer char formation [33,34,35]. The addition of Zn^2+^ has been reported to improve the flame-retardant performance of PA6 systems [36]. Therefore, utilizing the electrostatic interaction between transition metal ions and the phosphate groups of PA significantly catalyzes carbon formation, effectively suppressing the release of heat and smoke.

The purpose of this work is to overcome the insufficient condensed-phase charring efficiency and high smoke release of aluminum diethylphosphinate in polyamide 6. To this end, a synergistic flame-retardant system, denoted as PABG@Zn, was constructed by integrating a highly oxidized phosphorus source (phytic acid), a nitrogen-containing component (benzoguanamine), and a condensed-phase catalyst (Zn^2+^). The novelty of this work lies in the rational construction of a P/N/Zn cooperative flame-retardant system that simultaneously enhances condensed-phase char formation and suppresses smoke evolution at low additive loading in PA6/ADP composites, providing a novel strategy for achieving more efficient flame-retardant modification. The uniqueness of the conducted research lies in the evaluation of glow-wire ignition and electrical tracking resistance in addition to conventional flame-retardant tests, thereby providing a more comprehensive assessment of the material’s fire safety.

## 2. Experimental Section

### 2.1. Materials

Polyamide 6 (PA6, BASF SE, B30S,) was supplied by Honghua Plastics Technology (Dongguan, China). Aluminum diethyl hypophosphite (ADP, 99.5%) was obtained from Qingdao OPERATE New Material Co., Ltd., Qingdao, China. Phytic acid (70 wt%), benzoguanamine (BG, 98%), and zinc sulfate heptahydrate (ZnSO_4_·7H_2_O, 99.5%) were purchased from Macklin Biochemical Co., Ltd. (Shanghai, China). Deionized water was produced in the laboratory.

### 2.2. Synthesis of PABG

PABG was prepared via ionic bonding between PA and BG. To maximize the reaction between the P–OH groups of PA and the –NH_2_ groups of BG, a PA/BG molar ratio of 1:6 was employed. Briefly, 5.62 g of BG was dissolved in 400 mL of deionized water at 60 °C under continuous stirring. Separately, 4.71 g of PA solution (70 wt%) was diluted to 25 mL and then added dropwise to the BG solution using a constant-pressure funnel. The mixture was stirred at 80 °C for 2 h, during which a white precipitate gradually formed. The precipitate was collected by filtration, washed three times with hot water, and then dried at 80 °C for 24 h, yielding 8.03 g of the intermediate product with a yield of 90.1%, which was denoted as PABG.

### 2.3. Synthesis of PABG@Zn

**Figure 1:**
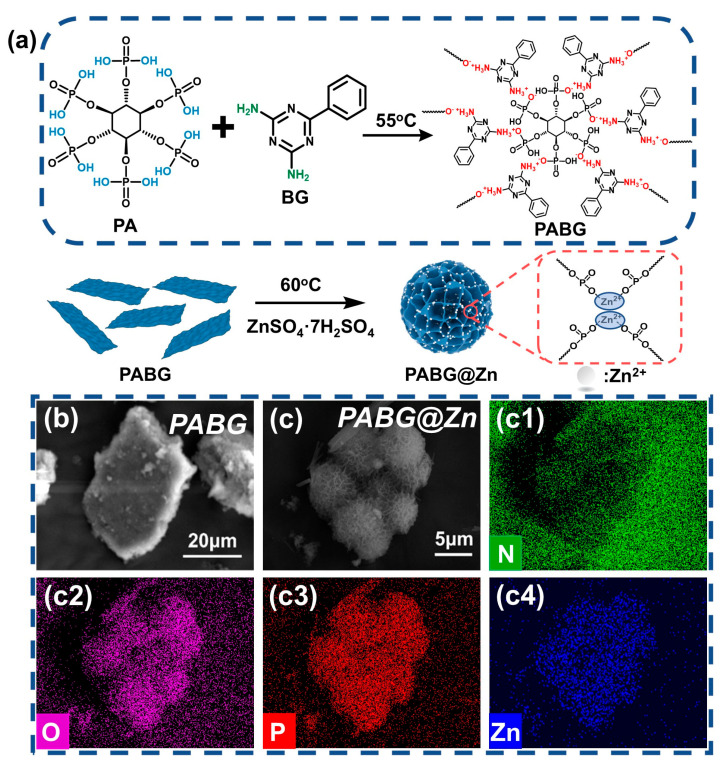
(**a**) Schematic of the PABG@Zn synthesis route, SEM images of (**b**) PABG and (**c**) PABG@Zn, and elemental mappings of (**c1**) N, (**c2**) O, (**c3**) P, and (**c4**) Zn in PABG@Zn.

Firstly, 5.35 g of PABG was dissolved in 200 mL of methanol under ambient conditions with continuous stirring. Subsequently, 2 g of ZnSO_4_·7H_2_O was slowly added to the PABG solution, and the mixture was stirred for 30 min to facilitate the coordination reaction. The resulting slurry was centrifuged three times with methanol and deionized water, respectively, to remove the unreacted species. The solid product was dried in a vacuum oven at 80 °C for 12 h and then ground to obtain 4.96 g of powder with a yield of 85.2%, designated as PABG@Zn. The synthesis route of the flame retardant is illustrated in Figure 1a.

### 2.4. Preparation of Flame-Retardant PA6 Composites

Prior to processing, the PA6 pellets were dried at 80 °C for 10 h. Flame-retardant PA6 composites containing varying ratios of additives were then prepared using a twin-screw extruder at a screw speed of 50 rpm, with three heating zones set at 220, 230, and 240 °C. The extrudates were pelletized using a cutter, and to ensure homogeneous dispersion of the flame retardants, the extrusion–pelletization process was repeated twice. The resulting pellets were subsequently melt-processed into standard specimens for testing.

All flame-retardant PA6 composites contained a total additive loading of 15 wt% and were designated as PA6/ADP_15_, PA6/ADP_12_/PABG_3_, PA6/ADP_10_/PABG@Zn_5_, PA6/ADP_12_/PABG@Zn_3_, PA6/ADP_13_/PABG@Zn_2_, and PA6/ADP_14_/PABG@Zn_1_, with the subscript numbers indicating the mass fraction of each component (detailed in Table 1). Pure PA6 specimens were prepared under the same processing conditions as controls.

### 2.5. Characterizations

Fourier-transform infrared (FTIR) spectra were recorded on a Nicolet iS5 spectrometer (Thermo Fisher, Waltham, MA, USA). Powder samples were prepared using the KBr pellet method and scanned over the range of 4000~500 cm^−1^ with a resolution of 4 cm^−1^ and 32 scans [37].

X-ray photoelectron spectroscopy (XPS) was performed on an ESCALAB 250Xi system (Thermo Fisher, Waltham, MA, USA) with Al Kα radiation as the X-ray source, a voltage of 284.8 eV, and a step size of 0.5 eV.

The morphology of the samples and the energy-dispersive X-ray spectroscopy (EDS) were examined by scanning electron microscopy (SEM) on a HITCHI S-4700 instrument (Hitachi, Tokyo, Japan) at an accelerating voltage of 5–15 kV. Prior to imaging, the samples were coated with a thin conductive layer.

Thermogravimetric analysis (TGA) was carried out on a TGA Q50 instrument (TA Instruments, Newcastle, WA, USA). Approximately 3–5 mg of sample was heated from 30 to 700 °C at a rate of 10 °C/min under a nitrogen atmosphere.

The limiting oxygen index (LOI) was measured using a JF-3 oxygen index tester (Nanjing Jiangning Analytical Instrument Co., Nanjing, China). Samples were prepared with dimensions of 100 × 6.5 × 3.2 mm^3^, and the reported values represent the average of five parallel specimens.

Vertical burning tests (UL-94) were conducted using a CZF-5 horizontal/vertical burning tester (Nanjing Jiangning Analytical Instrument Co., Nanjing, China). The sample dimensions were 100 × 13 × 3.2 mm^3^, with five specimens tested per group.

Glow-wire tests (GWIT/GWFI) were performed on a hot-wire tester (Suzhou Yangyi Walch Testing Technology Co., Jingsu, China). The samples were prepared with dimensions of 60 × 60 × 3.2 mm^3^, and three specimens were tested per temperature condition to determine the glow-wire ignition time and glow-wire flammability index.

Comparative tracking index (CTI) measurements were conducted using a corresponding tester (Suzhou Yangyi Walch Testing Technology Co., Jingsu, China). Samples of 20 × 20 × 3.2 mm^3^ were tested at each voltage, with five specimens per condition.

Cone calorimeter tests were performed using a VOUCH 6810 instrument (Suzhou Yangyi Walch Testing Technology Co., Jingsu, China). Samples measured 100 × 100 × 3.2 mm^3^ and were exposed to a heat flux of 50 kW/m^2^, and the key parameters including the heat release rate and smoke generation were recorded.

## 3. Results and Discussion

### 3.1. Structural Characterization of PABG@Zn

The typical synthetic route of PABG@Zn was illustrated in Figure 1a. The morphologies of PABG and PABG@Zn were characterized by SEM. As shown in Figure 1b, PABG exhibited an irregular micron-sized layered structure, indicating successful electrostatic self-assembly between PA and BG. Upon Zn^2+^ incorporation, PABG@Zn underwent significant morphological evolution, forming wrinkled microspheres with a porous honeycomb-like structure (Figure 1c). This transformation was primarily attributed to coordination interactions between Zn^2+^ and the P–OH groups, which reduced the surface energy and drove the two-dimensional layers into spherical architectures. EDS of PABG@Zn (Figure 1(c1–c4)) revealed uniform distributions of P, O, N, and Zn, preliminarily confirming the integrity of the PABG@Zn framework.

**Figure 2:**
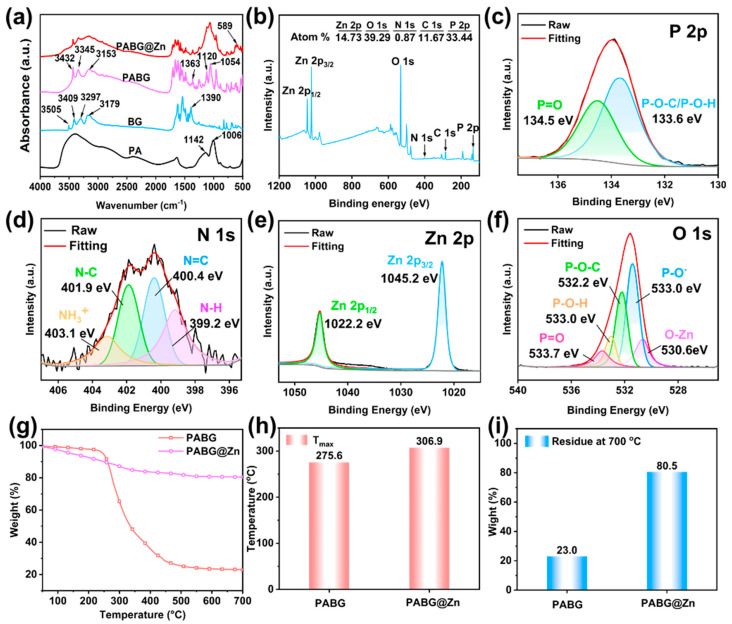
(**a**) FTIR spectra of PA, BG, PABG, and PABG@Zn; (**b**) XPS survey spectrum of PABG@Zn; high-resolution XPS spectra of (**c**) P2p, (**d**) N1s, (**e**) Zn2p, and (**f**) O1s of PABG@Zn; (**g**) TGA curves of PABG and PABG@Zn; (**h**) corresponding T_max_ and (**i**) char residue yield at 700 °C.

To elucidate the binding mechanism of PABG@Zn, the FTIR spectrum was employed to analyze changes in the functional groups (Figure 2a). In the PA spectrum, characteristic peaks of P–O–C and P=O appeared at 1142 and 1054 cm^−1^, respectively [38], while BG exhibited –NH_2_ stretching vibrations in the 3505–3179 cm^−1^ [39]. In PABG, the P=O and P–O–C peaks were shifted, and the –NH_2_ stretching vibrations underwent pronounced redshifts to 3432, 3345, and 3153 cm^−1^, with the C–N peak moving to 1363 cm^−1^ [40]. These changes indicated the formation of –NH_3_^+^ ions, confirming that PABG was assembled through electrostatic interactions between the phosphoric groups of PA and the amino groups of BG [41].

In the FTIR spectrum of PABG@Zn, a peak at 589 cm^−1^ corresponding to Zn–O was observed [42], directly indicating the coordination of Zn^2+^ with –OH groups. Further insights into the chemical bonding were obtained via XPS (Figure 2b–f). The survey spectrum (Figure 2b) confirmed the presence of C, P, O, N, and Zn, consistent with the EDS results. In the P2p spectrum (Figure 2c), P=O was observed at 134.5 eV, while P–O–C and P–O–H appeared at 133.6 eV [43]. The N1s spectrum (Figure 2d) revealed C–N (401.9 eV), C=N (400.4 eV), and –NH_3_^+^ (403.1 eV), in agreement with the FTIR findings [44]. The Zn2p spectrum (Figure 2e) displayed characteristic Zn2p_1/2_ and Zn2p_3/2_ peaks at 1022.2 and 1045.2 eV [45]. Additionally, the O1s spectrum (Figure 2f) showed O–Zn at 530.6 eV in addition to P=O, P–O–H, and P–O–C signals [46], providing direct evidence of coordination between Zn^2+^ and PABG.

The TGA results of PABG and PABG@Zn (Figure 2g and Table 2) showed that the introduction of Zn^2+^ markedly enhanced the thermal stability. As shown in Figure 2h, the maximum decomposition temperature (T_max_) of PABG@Zn increased significantly from 275.6 °C to 306.9 °C. More notably, the char-forming capability was improved: the char yield of PABG at 700 °C was only 23.0%, whereas PABG@Zn reached 80.5% (Figure 2i). Thus, Zn^2+^ coordination enhanced the thermal stability, meeting the high-temperature processing requirements of PA6.

### 3.2. Thermal Stability of Flame-Retardant PA6 Composites

**Figure 3:**
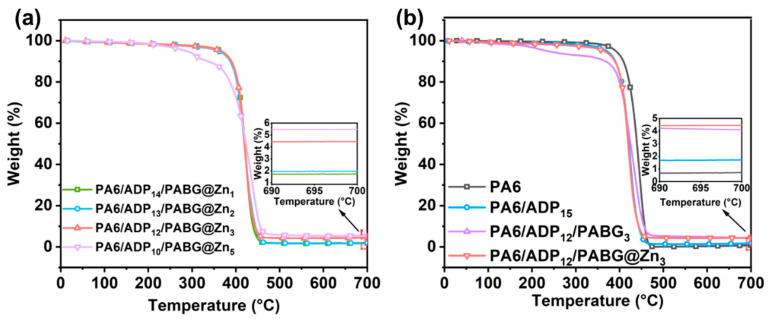
(**a**) TG curves of PABG@Zn replacing ADP at different loading levels under nitrogen atmosphere. (**b**) TGA curves of PA6 and FRPA6 composites.

Figure 3a and Table 2 compare the thermal degradation behaviors of flame-retardant composites to determine the optimal substitution ratio of PABG@Zn. As PABG@Zn gradually replaced ADP, the char residue increased accordingly, and a more pronounced charring effect was observed at a substitution level of 3%, while the temperature at 5% weight loss (T_5_%) remained well above the processing temperature of PA6 (240 °C). However, when the substitution level was increased to 5% (PA6/ADP_10_/PABG@Zn_5_), the T_5_% sharply decreased to 286.7 °C, approaching the processing window of PA6. Based on the above analysis, when ADP was replaced by PABG@Zn at a ratio of 3%, it achieved an excellent balance between thermal stability and carbonization performance, making 3% the optimal addition amount, which was retained for further study of the flame retardancy of FRPA6.

Figure 3b and Table 2 present the TGA curves of PA6 and flame-retardant PA6 (FRPA6) under a nitrogen atmosphere. Compared with the control PA6, all FRPA6 samples show slightly lower temperatures at 5% weight loss (T_5_%), which can be attributed to the early decomposition of the flame retardants, enabling the formation of a protective barrier before severe degradation of the polymer matrix. In addition, control PA6 exhibits a low char yield of only 1.7% at 700 °C, and even with the addition of 15 wt% ADP, the char yield increases only marginally to 2.0%, indicating the limited condensed-phase flame-retardant effect of the conventional ADP system. Upon introducing PABG, the char yield increases to 4.1%, which is attributed to the formation of phosphorus-containing species during high-temperature decomposition that promote dehydration carbonization of the matrix. With further incorporation of PABG@Zn, the char residue increases to 4.6%, suggesting that the metal complex enhances the dehydration carbonization efficiency through a synergistic catalytic effect.

### 3.3. Flame Retardancy

**Figure 4:**
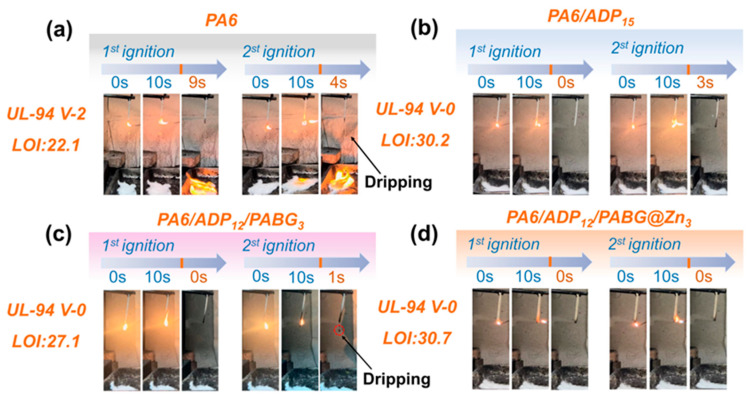
Digital photos of (**a**) control PA6, (**b**) PA6/ADP_15_, (**c**) PA6/ADP_12_/PABG_3_, and (**d**) PA6/ADP_12_/PABG@Zn_3_ during the UL-94 vertical burning tests.

As shown in Figure 4, the burning times (t1, t2), UL-94 ratings, and LOI values of PA6 and its flame-retardant composites were evaluated. The control PA6 ignited readily and exhibited intense melt-dripping that ignited the underlying cotton, achieving only a UL-94 V-2 rating with an LOI of 22.1%, demonstrating insufficient inherent flame resistance. Introducing 15% ADP (PA6/ADP_15_) significantly enhanced the flame retardancy of PA6, enabling a UL-94 V-0 rating and increasing the LOI to 30.2%.

However, in the PA6/ADP_12_/PABG_3_ formulation, noticeable melt-dripping persisted during combustion. Although the drips no longer ignited the cotton, the UL-94 rating decreased to V-1, and the LOI (27.1%) also declined. In contrast, the PA6/ADP_12_/PABG@Zn_3_ system exhibited an outstanding flame-retardant ability. The material self-extinguished rapidly after ignition, while achieving the highest LOI of 30.7%.

**Figure 5:**
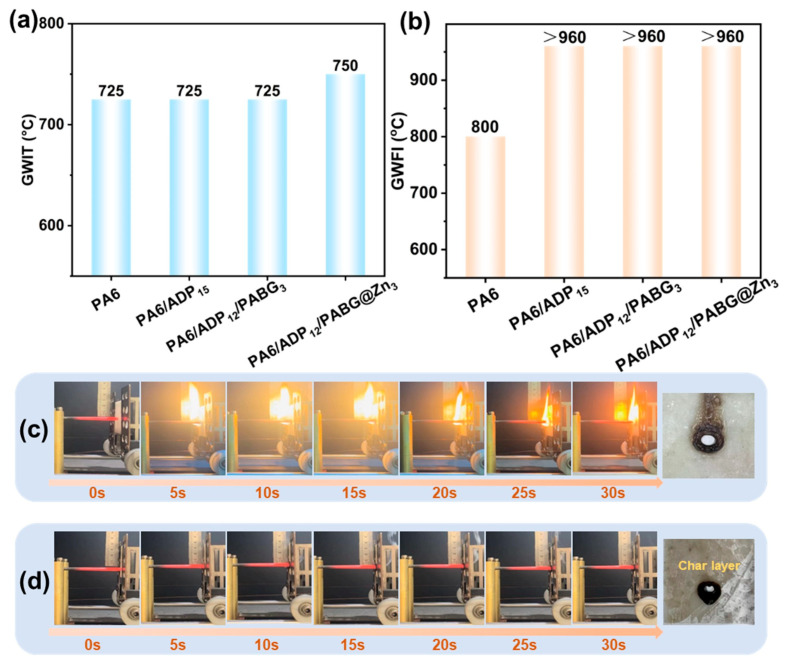
(**a**) GWIT and (**b**) GWFI results of control PA6 and flame-retardant PA6; the glow-wire test (750 °C) char residue formation of (**c**) PA6/ADP_15_ and (**d**) PA6/ADP_12_/PABG@Zn_3_.

Aside from open flames, localized overheating caused by short circuits is also a critical ignition source in electrical fires. Therefore, glow-wire tests were employed to assess the high-temperature resistance and flame-retardant reliability of the materials. The key results are summarized in Figure 5 and Table 3 and Table 4.

As shown in Figure 5a,b, the control PA6 exhibited a low glow-wire ignition temperature (GWIT) of only 725°C and a glow-wire flammability index (GWFI) of 800 °C, failing to meet the safety requirements for electrical components under thermal shock (GWIT > 750 °C, GWFI > 850 °C). Upon incorporation of flame retardants, all systems achieved the GWFI of “>960 °C”, indicating that the samples self-extinguished within 30 s after the 960 °C glow-wire was withdrawn. However, for the GWIT values, only the PA6/ADP_12_/PABG@Zn_3_ showed an elevated GWIT (750 °C), highlighting its superior resistance to ignition under high-temperature conditions. Compared with mica mineral [47] and tri-hydrated aluminum [48], which are commonly used to improve glow-wire temperature in the literature, PABG@Zn can achieve comparable or better performance at a lower overall loading.

Figure 5c,d compare the glow-wire test responses of PA6/ADP_15_ and PA6/ADP_12_/PABG@Zn_3_ at 750 °C. PA6/ADP_15_ exhibited limited charring efficiency and insufficient flame suppression, igniting rapidly upon contact with the glow wire. In contrast, PA6/ADP_12_/PABG@Zn_3_ did not ignite under the same conditions and developed a thicker more continuous char layer. This demonstrated that PABG@Zn effectively promoted the rapid formation of a dense carbon barrier, enabling superior glow-wire resistance.

Furthermore, electrical applications place strict demands on insulation performance. To evaluate whether introducing Zn^2+^ from PABG@Zn affected the insulation properties, tracking resistance tests were conducted. The control PA6 exhibited a comparative tracking index (CTI) of 600 V; this value slightly decreased to 550 V with 15% ADP and remained at 550 V after incorporating PABG@Zn. These results confirmed that the addition of the PABG@Zn did not influence the insulation performance of the PA6/ADP system.

**Figure 6:**
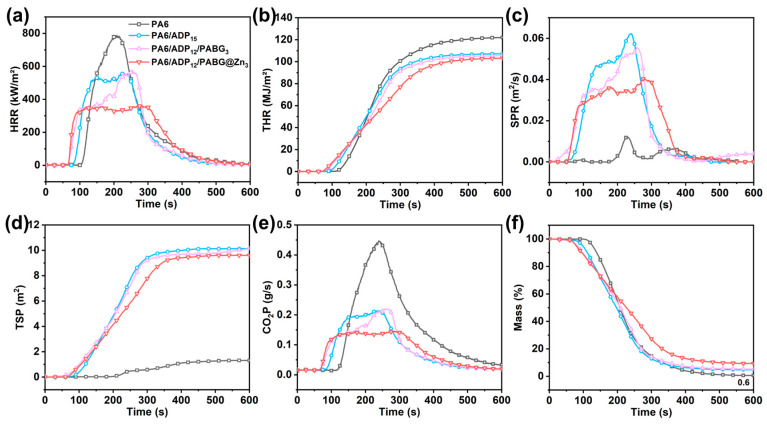
Cone calorimetry results of PA6 and its flame-retardant composites: (**a**) pHRR, (**b**) THR, (**c**) SPR, (**d**) TSP, (**e**) CO_2_ production rate, and (**f**) mass loss curves.

Cone calorimetry was employed to simulate the combustion behavior of the materials under realistic fire conditions. The key results for PA6 and its flame-retardant composites are summarized in Figure 6 and Table 5 and Table 6. The control PA6 exhibited typical high flammability, with a peak heat release rate (pHRR) of 787.3 kW/m^2^ and a total heat release (THR) of 122.0 MJ/m^2^ (Figure 6a,b), indicating a substantial fire hazard. Incorporation of 15% ADP significantly reduced the pHRR to 549.0 kW/m^2^ and the THR to 107.3 MJ/m^2^. Notably, the PA6/ADP_12_/PABG@Zn_3_ achieved a pHRR of 362.4 kW/m^2^ and THR of 103.1 MJ/m^2^, corresponding to reductions of 34.0% and 4.1% relative to PA6/ADP_15_. Smoke hazards often pose a larger threat than flames during fires; therefore, the smoke-suppressing performance of the flame-retardant systems was further evaluated via the total smoke production (TSP), smoke production rate (SPR), and CO_2_ production rate (CO_2_P) [49]. As shown in Figure 6c,e, PA6/ADP_15_ generally generated considerable smoke, due to its predominant gas-phase flame-retardant mechanism. Notably, PA6/ADP_12_/PABG@Zn_3_ exhibited reductions of 33.3%, 5.0%, and 33.3% in SPR, TSP, and CO_2_P, respectively, compared to PA6/ADP_15_. The mass loss curves (Figure 6f) further confirmed the condensed-phase flame-retardant behavior. The control PA6 left only 0.6% residue, while PA6/ADP_15_ and PA6/ADP_12_/PABG_3_ yield 4.7% and 5.2% char, respectively, reflecting the limited charring ability of ADP and PABG. In contrast, PA6/ADP_12_/PABG@Zn_3_ achieved a char residue of 9.6%.

TTI refers to the time to ignition, i.e., the duration from the onset of heat flux on the material surface to the moment when sustained combustion occurs. For the control PA6, the TTI was 102 s. The TTI of PA6/ADP_15_, PA6/ADP_12_/PABG_3_, and PA6/ADP_12_/PABG@Zn_3_ was shortened, which could be attributed to the fact that the flame retardants (e.g., ADP, PABG, PABG@Zn) decomposed earlier than the polymer matrix, forming a protective char layer that enhanced the overall flame retardancy. This behavior was consistent with the results in Section 3.2, where the addition of flame retardants also caused the temperature at 5% mass loss (T_5_%) to occur earlier.

The fire safety of the materials was assessed using the Fire Performance Index (FPI) and Fire Growth Index (FGI). The FPI and FGI were calculated by Formulas (1) and (2) based on the cone calorimeter test results presented in Table 5, following the previously reported methods [50,51], and the corresponding values are summarized in Table 6. Higher FPI values indicate lower fire risk, while lower FGI values reflect slower fire spread. Among all the samples, PA6/ADP_12_/PABG@Zn_3_ exhibited the highest FPI (0.17 kW·m^−2^·s^−1^) and the lowest FGI (1.29 m^2^·s·kW^−1^), indicating the lowest ignition propensity and the slowest flame spread rate and, thus, superior fire safety.

To further verify the flame-retardant efficiency in both the gas phase and condensed phase, the results shown in Table 5 were subjected to quantitative analysis by calculating the flame inhibition, charring effect, and barrier-protective effect by Formulas (3)–(5), according to the previously reported methods [52]. The flame inhibition reflects the gas-phase flame-retardant efficiency, with higher values indicating stronger gas-phase inhibition. The charring effect and barrier-protective effect represent the condensed-phase flame-retardant efficiency, where higher values indicate more effective condensed-phase protection. PA6/ADP_12_/PABG@Zn_3_ exhibited a flame inhibition value of 0.16%, which was slightly lower than that of PA6/ADP_15_, indicating a modest reduction in gas-phase activity. However, PA6/ADP_12_/PABG@Zn_3_ exhibited enhanced condensed-phase performance, achieving a charring effect of 8.8% and a barrier-protective effect of 45.6%, compared with the limited values of 4.1% and 20.8% observed for PA6/ADP_15_. This trend was consistent with the thermal shielding observed in the glow-wire testing, further confirming the dominant role of PABG@Zn in promoting condensed-phase flame retardancy.

(1) $$\mathrm{FPI}=\frac{\mathrm{TTI}}{\mathrm{pHRR}}$$

(2) $$\mathrm{FGI}=\frac{\mathrm{pHRR}}{\mathrm{Time}\;\mathrm{to}\;\mathrm{reach}\;\mathrm{pHRR}}\;$$

(3) $$\mathrm{Flame}\;\mathrm{inhibition}=1-\frac{{\mathrm{EHC}}_{\mathrm{FRPA}6}}{{\mathrm{EHC}}_{\mathrm{PA}6}}$$

(4) $$\mathrm{Charring}\;\mathrm{effect}=\frac{1-{\mathrm{TML}}_{\mathrm{FRPA}6}}{{\mathrm{TML}}_{\mathrm{PA}6}}$$

(5) $$\mathrm{Barrier}\text{-}\mathrm{protective}\;\mathrm{effect}=\frac{1-({\mathrm{pHRR}}_{\mathrm{FRPA}6}/{\mathrm{pHRR}}_{\mathrm{PA}6})}{{\mathrm{THR}}_{\mathrm{FRPA}6}/{\mathrm{THR}}_{\mathrm{PA}6}}$$

where TML represents the total mass loss during the cone calorimetry test.

### 3.4. Fire-Retardant Mechanism

**Figure 7:**
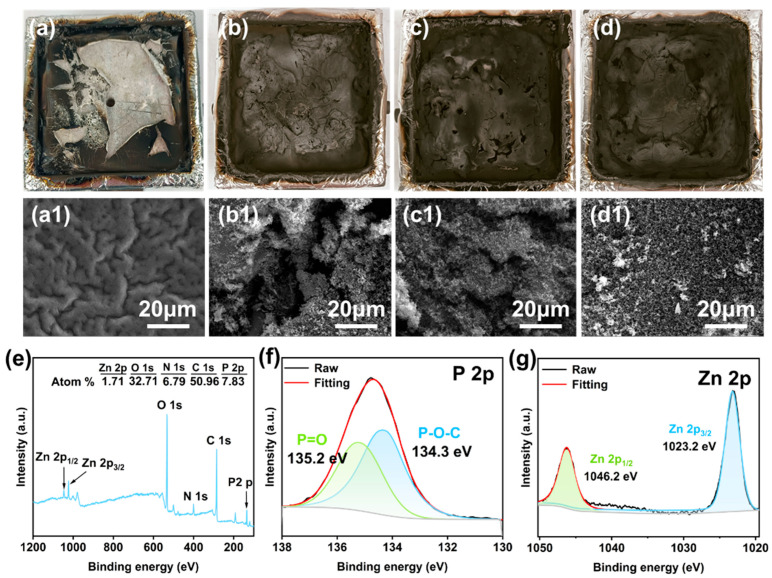
Photographs of the residual carbon layers after cone calorimeter testing: (**a**) PA6 (**a1**) 20 μm, (**b**) PA6/ADP_15_ (**b1**) 20 μm, (**c**) PA6/ADP_12_/PABG_3_ (**c1**) 20 μm, and (**d**) PA6/ADP_12_/PABG@Zn_3_ (**d1**) 20 μm; (**e**) XPS survey spectra of char residue from PA6/ADP_12_/PABG@Zn_3_; high resolution XPS spectra of (**f**) P2p and (**g**) Zn2p spectra for PA6/ADP_12_/PABG_3_ char residue.

To further elucidate the effect of PABG@Zn on the PA6 combustion behavior, the char morphologies of each system after cone calorimetry were examined via SEM (Figure 7a–d). The control PA6 formed almost no expanded structure, with only fragment residue. In contrast, PA6/ADP_15_ and PA6/ADP_12_/PABG_3_ generated partially expanded char layers, yet their surfaces displayed numerous irregular microcracks, providing pathways for the escape of pyrolysis volatiles and the entry of external combustible gases. Notably, PA6/ADP_12_/PABG@Zn_3_ developed a more continuous and morphologically stable char layer (Figure 7d), effectively enhancing the char shielding effect by blocking heat and volatile transfer.

To further illustrate the formation mechanism of the dense char layer, XPS characterization was conducted on the residues. As shown in Figure 7e, pronounced P2p and Zn2p signals were detected in the PA6/ADP_12_/PABG@Zn_3_ char, indicating the migration and enrichment of P and Zn species at the char surface during combustion, which facilitated the construction of a stable inorganic–organic hybrid char network. The high-resolution P2p spectra (Figure 7f) revealed characteristic P=O (135.2 eV) and P–O–C (134.3 eV) bonding [53], suggesting that phosphorus-containing intermediates generated during PABG@Zn pyrolysis. Concurrently, Zn2p analysis (Figure 7g) confirmed the presence of Zn (1046.2 eV and 1023.2 eV) [54], demonstrating its presence in the condensed phase, which benefits the formation of a dense, continuous, and protective char layer.

**Figure 8:**
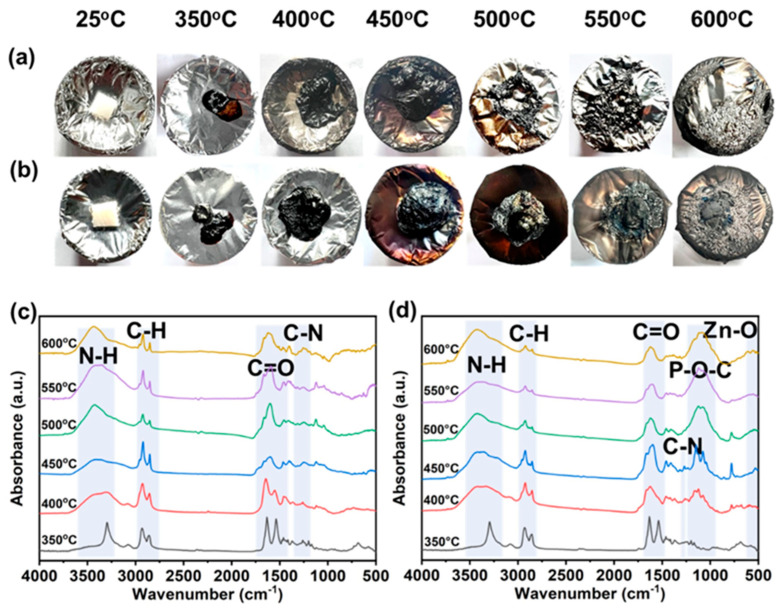
Char residue obtained from muffle furnace tests for (**a**) control PA6 and (**b**) PA6/ADP_12_/PABG@Zn_3_; FTIR spectra of the corresponding char residue at various temperatures for (**c**) control PA6 and (**d**) PA6/ADP_12_/PABG@Zn_3_.

To clarify the condensed-phase flame-retardant mechanism of PABG@Zn in PA6, PA6 and PA6/ADP_12_/PABG@Zn_3_ were treated in a muffle furnace, and the char residues at different temperatures were investigated by FTIR analysis. As shown in Figure 8a,b, the difference in the charring capability between the two systems was evident. For control PA6, the residues rapidly shrank and fragmented with increasing temperature, leaving minimal char at 600 °C, indicating poor thermal stability with a fast and complete pyrolysis process. In contrast, PA6/ADP_12_/PABG@Zn_3_ exhibited significantly enhanced charring ability and structural integrity across the entire temperature range. Notably, even at 600 °C, PA6/ADP_12_/PABG@Zn_3_ retained a larger and more intact char, effectively improving the charring efficiency of PA6.

The FTIR spectra of the char residue from the control PA6 primarily exhibited characteristic absorptions of the PA6 molecular backbone: N–H stretching at 3440–3298 cm^−1^, C–H stretching at 2927–2855 cm^−1^, C=O stretching at 1638–1537 cm^−1^, and C–N stretching at 1269–1244 cm^−1^ [55]. As the thermal treatment temperature increased from 350 °C to 600 °C, the intensities of all these peaks continuously and rapidly decreased, indicating that the PA6 backbone underwent pronounced thermal decomposition and depolymerization. This process progressively degraded the carbon structure, ultimately yielding predominantly low-molecular-weight volatile products.

In contrast, the FTIR spectra of PA6/ADP_12_/PABG@Zn_3_ char retained N–H, C–H, C=O, and C–N signals even at 600 °C. Although their intensities decreased, they remained substantially stronger than those of the control PA6, suggesting that the primary thermal decomposition pathways were similar to PA6, but the degradation rate of the PA6 backbone was effectively suppressed. Notably, the broad band at 1077~1163 cm^−1^ corresponded to phosphate ester or polyphosphate structures [56,57], indicating that phosphorus-containing derivatives generated from PABG@Zn underwent catalytic dehydration and chemical crosslinking with the PA6 pyrolysis products, promoting the formation of a stable P–O–C-rich crosslinked char. The new peak at 536–553 cm^−1^ was attributed to Zn species converting to stable ZnO at high temperature [58], with the corresponding Zn–O signal further confirming its catalytic role in condensed-phase char formation.

**Figure 9:**
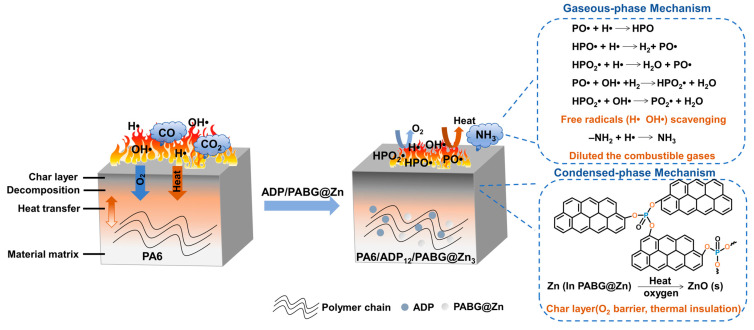
Schematic illustration of the flame-retardant mechanism of PA6/ADP_12_/PABG@Zn_3_.

Based on the comprehensive results, the flame-retardant mechanism of PA6/ADP_12_/PABG@Zn_3_ is illustrated in Figure 9. Upon heating, the PA6 matrix underwent thermal decomposition, generating reactive radicals (HO•, H•), hydrocarbons, and volatile species such as CO_2_ and CO. In the gas phase, phosphorus-containing radicals (PO•, HPO•, HPO_2_•) released from ADP effectively scavenged these high-energy radicals, thereby suppressing the chain reactions of combustion. Simultaneously, NH_3_ decomposed from the BG in PABG@Zn diluted the combustible gases, further reducing the flame intensity.

In the condensed phase, the PA groups in PABG@Zn formed phosphorus-containing species at elevated temperatures, catalyzing PA6 dehydration and char formation, which promoted the development of a dense P–O–C-rich char layer. Concurrently, Zn ions converted to stable ZnO under high temperature, participating in the catalytic charring process to accelerate char layer growth and enhance its density. The resulting continuous char effectively blocked heat and oxygen transfer, thereby inhibiting further matrix degradation and combustion. Overall, the superior flame retardancy of PA6/ADP_12_/PABG@Zn_3_ originated from the synergistic action of the flame-retardant system in both the gas and condensed phases.

## 4. Conclusions

This work demonstrated that integrating a synergist (PABG@Zn) into PA6/ADP systems provided a highly effective route to reinforce condensed-phase protection and overall fire safety without compromising the intrinsic processing characteristics of the matrix. Benefiting from the P–N–Zn synergistic effect, PABG@Zn significantly accelerated the carbonization kinetics of the PA6/ADP matrix. With only a 3% replacement of ADP, the optimized composite (PA6/ADP_12_/PABG@Zn_3_) presented a strengthened condensed-phase response, reflected by an increased char yield (9.3%) and pronounced reductions in pHRR and SPR (−34.0% and −33.3%, respectively), surpassing the performance of the conventional PA6/ADP_15_ system. More importantly, the rapidly formed dense carbon layer prevents heat transfer, allowing the composite material to meet electrical safety requirements, achieving a glow-wire ignition temperature (GWIT) of 750 °C and the highest glow-wire flammability index (GWFI) rating (>960 °C). Considering the low loading level and the widespread industrial use of zinc compounds, the incorporation of PABG@Zn is not expected to introduce significant additional environmental or economic burdens. Overall, PABG@Zn served as an efficient condensed-phase promoter for PA6/ADP composites, offering a practical strategy to simultaneously improve the flame retardancy, charring efficiency, and glow-wire safety performance.

## Figures and Tables

**Table 1:** The formula of flame-retardant PA6 composites.

Sample	PA6 (g)	ADP (g)	PABG (g)	PABG@Zn (g)
PA6	100	-	-	-
PA6/ADP_15_	85	15	-	-
PA6/ADP_12_/PABG_3_	85	12	3	-
PA6/ADP_10_/PABG@Zn_5_	85	10	-	5
PA6/ADP_12_/PABG@Zn_3_	85	12	-	3
PA6/ADP_13_/PABG@Zn_2_	85	13	-	2
PA6/ADP_14_/PABG@Zn_1_	85	14	-	1

**Table 2:** The key data of TGA tests.

Samples	T_5%_ (°C)	T_max_ (°C)	MLR_max_ (%/°C)	Theoretical Residue (%)	Residue (%)
PA6	391.2	446.2	2.5	/	1.7
ADP	388.5	450.1	2.0	/	9.3
PABG	246.9	275.6	0.8	/	23.0
PABG@Zn	201.5	305.9	0.1	/	80.5
PA6/ADP_15_	369.4	423.0	2.6	2.8	2.0
PA6/ADP_12_/PABG_3_	231.7	405.3	2.6	3.3	4.1
PA6/ADP_12_/PABG@Zn_3_	364.1	420.0	2.6	5.0	4.6
PA6/ADP_14_/PABG@Zn_1_	366.2	422.8	2.8	3.6	1.7
PA6/ADP_13_/PABG@Zn_2_	360.1	422.7	2.2	4.3	2.0
PA6/ADP_10_/PABG@Zn_5_	286.7	436.6	1.4	6.4	5.5

**Table 3:** State of control PA6 and PA6 composites at glow-wire ignition temperature (GWIT).

Samples (3.2 mm)	GWIT (°C)	Ignition/t_I_ (s)	Extinguishment/t_E_ (s)	t_E_ − t_R_ /t_T_ (s)	Specified Layers Ignited
PA6	725	0	0	0	N
PA6/ADP_15_	725	0	0	0	N
PA6/ADP_12_/PABG_3_	725	0	0	0	N
PA6/ADP_12_/PABG@Zn_3_	750	0	0	0	N

**Table 4:** State of control PA6 and PA6 composites at glow-wire flammability index (GWFI).

Samples (3.2 mm)	GWFI (°C)	Ignition/t_I_ (s)	Extinguishment/t_E_ (s)	t_E_ −30 /t_R_ (s)	Specified Layers Ignited
PA6	800	0.1	31.5	1.5	N
PA6/ADP_15_	960	0.4	32.6	2.6	N
PA6/ADP_12_/PABG_3_	960	0.2	31.3	1.3	N
PA6/ADP_12_/PABG@Zn_3_	960	0.4	31.5	1.5	N

**Table 5:** The key data of PA6 and its flame-retardant composites from cone calorimeter tests.

Samples	TTI (s)	EHC (MJ/kg)	pHRR (kW/m^2^)	THR (MJ/m^2^)	SPR (m^2^/s)	TSP (m^2^)	CO_2_P (g/s)	Residue (wt%)
PA6	102	33.16	787.3	122	0.01	1.31	0.46	0.6
PA6/ADP_15_	77	27.76	549.0	107.3	0.06	10.1	0.21	4.7
PA6/ADP_12_/PABG_3_	61	30.04	575.7	105.4	0.06	10.1	0.22	5.2
PA6/ADP_12_/PABG@Zn_3_	63	30.38	362.4	103.1	0.04	9.6	0.14	9.3

**Table 6:** Fire performances of PA6 and its flame-retardant composites.

Samples	FPI	FGI	Flame Inhibition (%)	Charring Effect (%)	Barrier-Protective Effect (%)
PA6	0.13	3.79	**/**	**/**	**/**
PA6/ADP_15_	0.14	2.34	0.16	4.1	20.8
PA6/ADP_12_/PABG_3_	0.11	2.26	0.09	4.6	15.4
PA6/ADP_12_/PABG@Zn_3_	0.17	1.29	0.08	8.8	45.6

## Data Availability

The original contributions presented in this study are included in the article. Further inquiries can be directed to the corresponding authors.
